# Association of Neutrophil-to-Lymphocyte Ratio and Lymphocyte-to-Monocyte Ratio with Treatment Modalities of Acute Ischaemic Stroke: A Pilot Study

**DOI:** 10.3390/medicina55070342

**Published:** 2019-07-05

**Authors:** Milena Świtońska, Artur Słomka, Piotr Korbal, Natalia Piekuś-Słomka, Władysław Sinkiewicz, Paweł Sokal, Ewa Żekanowska

**Affiliations:** 1 Department of Neurosurgery and Neurology, Nicolaus Copernicus University in Toruń, Ludwik Rydygier Collegium Medicum, 85-168 Bydgoszcz, Poland; 2 Department of Pathophysiology, Nicolaus Copernicus University in Toruń, Ludwik Rydygier Collegium Medicum, 85-094 Bydgoszcz, Poland; 3 Department of Propedeutics of Medicine, Nicolaus Copernicus University in Toruń, Ludwik Rydygier Collegium Medicum, 85-094 Bydgoszcz, Poland; 4 Department of Inorganic and Analytical Chemistry, Nicolaus Copernicus University in Toruń, Ludwik Rydygier Collegium Medicum, 85-089 Bydgoszcz, Poland; 5 2^nd^ Department of Cardiology, Nicolaus Copernicus University in Toruń, Ludwik Rydygier Collegium Medicum, 85-168 Bydgoszcz, Poland

**Keywords:** neutrophil-to-lymphocyte ratio, lymphocyte-to-monocyte ratio, ischaemic stroke, thrombectomy

## Abstract

*Background and Objectives:* Ischaemic stroke (IS) is the leading cause of death and disability worldwide. All stages of cerebral ischaemia, but especially acute phase, are associated with inflammatory response. Recent studies showed that neutrophil-to-lymphocyte ratio (NLR) and lymphocyte-to-monocyte ratio (LMR) may be used to assess inflammation in IS. To test whether there is a relationship between these parameters and type of stroke treatment, we analysed NLR and LMR in IS patients treated with three different modalities. *Materials and Methods:* The study included 58 adults with acute IS. A total of 28 patients received intravenous thrombolysis. In another 10 patients, the thrombolytic therapy was followed by thrombectomy and 20 patients did not undergo causal treatment. Blood samples were obtained within 24 h of the stroke diagnosis to calculate NLR and LMR. Next, NLR and LMR of the study subgroups were compared. *Results:* Our study revealed that NLR was significantly higher in patients treated with thrombectomy following thrombolysis, compared to no causal treatment. Statistical analysis demonstrated that patients with high National Institutes of Health Stroke Scale (NIHSS) scores presented higher NLR than in those with low NIHSS scores. Additionally, patients with high-sensitivity C-reactive protein (hs-CRP) ≥ 3 mg/L presented with significantly higher NLR and significantly lower LMR than the group of patients with lower hs-CRP (<3 mg/L). *Conclusions:* The main finding of this pilot study was that NLR in IS patients treated using thrombectomy following thrombolysis was markedly higher than that in other treatment groups, which was associated with increased severity of the disease in these patients. Therefore, patients with higher NLR may be expected to have more severe stroke. The link between stroke severity and NLR deserves further study.

## 1. Introduction

Stroke is the second leading cause of mortality and the first cause of disability in adults after myocardial infarction. Globally, ischaemic stroke accounts for 80–85% of all strokes. In Poland, over 90% of strokes are of ischaemic type [1]. Despite the prevalence of ischaemic stroke, its causal treatment still remains a challenge. The current available causal treatment of acute ischaemic stroke, including intravenous thrombolysis and mechanical thrombectomy, aims for recanalization of the cerebral arteries closed by an embolus or a clot. This results in the reperfusion of the hypoxic brain tissue.

It is well known that inflammatory reactions accompany all stages of cerebral ischaemia [2]. Ischaemic brain tissues release strongly pro-inflammatory chemokines which activate leukocytes and enhance their trans-endothelial migration to the site of inflammation [3]. This is an excellent example of the vicious circle which may be a target for a new neuroprotective treatment [4,5]. Of note, among leukocytes, neutrophils are a crucial mediator of the inflammatory response. A growing body of clinical evidence suggests that the neutrophil-lymphocyte ratio (NLR) can be used as an inexpensive and readily available marker to assess inflammation, especially in ischaemic stroke [6,7]. Moreover, NLR has proven to be associated with an increased risk of stroke severity and worse outcomes of patients diagnosed with ischaemic stroke [8,9]. Moreover, the results of a recent meta-analysis have demonstrated that a higher NLR is also related with an increased risk of secondary haemorrhage after ischaemic stroke [10]. Nonetheless, relatively little is known about the relationship between NLR, LMR and the therapeutic options in ischaemic stroke. Thus, the present preliminary report investigated whether NLR and LMR differ between three treatment groups of acute ischemic stroke patients: treated with thrombolysis, treated with thrombectomy following thrombolysis, and those who did not undergo any causal treatment. NLR and LMR may then be used as tools to examine whether there is a link between inflammation and stroke treatment options. Additionally, in the present study we aimed to explore the relationship between NLR, LMR and clinical outcome of stroke patients. Specifically, we decided to explore the association between both ratios, stroke scales and stroke classifications.

## 2. Materials and Methods

### 2.1. Subjects

This prospective study was conducted during the period 2017 to 2018 at the Stroke Intervention Treatment Centre, Department of Neurology, Jan Biziel University Hospital No. 2 in Bydgoszcz, Poland. The inclusion criteria were 18 or more years of age, acute ischaemic stroke [11] and no history of leukaemia and other cancer. The exclusion criteria were haemorrhagic stroke, transient ischemic attack (TIA) or a recent history of myocardial infarction (MI ≤ 1 month prior to the study), additional neurological disorders, clinical evidence of infection, liver and kidney failure, oral steroid therapy, pregnancy and inability to provide informed consent. The diagnosis of stroke was always made by the same experienced neurologist (MŚ) and confirmed using computed tomography (CT) and magnetic resonance imaging (MRI). The defined comorbidities and risk factors included a history of hypertension, atrial fibrillation (AF), diabetes mellitus, smoking, coronary artery disease (CAD) and dyslipidaemia. All comorbidities were defined according to previously established diagnosis. A history of stroke was also documented. Each patient was evaluated using the Trial of Org 10172 in Acute Stroke Treatment (TOAST) and the Oxfordshire Community Stroke Project (OCSP) classification systems [12,13]. The TOAST subtypes are large artery atherosclerosis, cardioembolism, small vessel occlusion, stroke of other determined aetiology and stroke of undetermined aetiology. The OCSP classification includes: total anterior circulation infarction (TACI), partial anterior circulation infarction (PACI) and lacunar infarction (LACI). For each patient, the following stroke scales were collected: National Institutes of Health Stroke Scale (NIHSS) at admission before treatment, NIHSS on the first day after treatment, modified Rankin Scale (mRS) at admission before treatment, 90-day mRS, and Alberta Stroke Program Early CT Score (ASPECTS) at admission before treatment. Grade of recanalization according to the thrombolysis in cerebral ischemia (TICI) grading system was assessed in patients treated with thrombectomy following thrombolysis. NIHSS on the first day after treatment was defined as NIHSS measured within 24 h after thrombolysis or thrombolysis following thrombectomy or NIHSS measured within 24 h after admission and before antiplatelet drug administration in the group of stroke patients not treated causally. These results of NIHSS evaluation were used in the analysis of the association between NLR, LMR and stroke severity.

Intravenous recombinant tissue plasminogen activator (rtPA, alteplase) was administered to 28 patients, endovascular thrombectomy following systemic thrombolytic therapy was conducted in 10 patients, while neither thrombolysis nor thrombectomy were used in 20 patients due to contraindications. The latter group received acetylsalicylic acid (ASA) at a daily dose of 150 mg. Thrombectomy was performed by the same interventional radiologist in patients with large-artery occlusion in anterior circulation. Patients received thrombolysis and thrombectomy in accordance with the criteria set out in the American Heart Association/American Stroke Association Guidelines [14]. The protocol of the study was reviewed and approved by the Local Bioethics Committee at Ludwik Rydygier Collegium Medicum, Nicolaus Copernicus University (Bydgoszcz, Poland, approval number: KB 694/2016, date of approval: 22 November 2016), and all participants signed the informed consent form.

### 2.2. Methods

Ethylenediaminetetraacetic acid (EDTA) blood samples were taken from patients within 24 h after interventions. Patients who did not undergo causal treatment had their blood samples taken within 24 h after stroke diagnosis and before ASA administration. The time point was chosen on the basis of a previous paper [15]. Complete blood count (CBC), including white blood cell (WBC) count, neutrophil count, lymphocyte count and monocyte count, was determined using an automated blood counter (XT-4000i, Sysmex Corporation, Kobe, Japan). NLR was calculated as the ratio of the number of neutrophils to the number of lymphocytes. Similarly, LMR was calculated by dividing the number of lymphocytes by the number of monocytes. Other biochemical parameters were measured using standard methods at central hospital laboratory.

### 2.3. Data Analysis

All data were analysed with the use of Statistica software, version 13.4 (Dell Computer Corporation, Round Rock, TX, USA). Categorical variables were compared with the use of the Pearson’s chi-square tests and are reported as percentages and frequencies. The normal distribution of each continuous variable was assessed with the use of the Shapiro-Wilk tests and they were analysed with the use of the Kruskal-Wallis tests or the Mann-Whitney *U* tests and are reported as medians and interquartile ranges. Association between continuous variables was expressed by the Pearson correlation coefficient. Differences were considered statistically significant if corresponding *p* values were below 0.05.

## 3. Results

### 3.1. Demographic and Clinical Data of the Study Group

Of the 59 patients screened, one female patient was not included in the analysis due to a hemicraniectomy after a severe stroke. Thus, the final sample size for this study was 58 IS patients, including 28 thrombolytic patients, 10 patients who underwent thrombectomy following thrombolysis, and 20 patients who did not receive any causal treatment. There were 29 women and 29 men, making the sex ratio 1:1. The median age of patients was 67 years (IQR, 60–77 years). Overall, 45 patients had hypertension, 16 had diabetes mellitus, 32 had dyslipidaemia, 11 had AF, 10 had CAD, 12 had a history of ischaemic stroke, and 18 were current smokers. As shown in Table 1, there were no significant differences between patients from the three therapeutic subgroups in terms of age, sex, body mass index (BMI), stroke classifications (TOAST and OSCP), left ventricular ejection fraction (LVEF), liver function, including aspartate aminotransferase (AST) and alanine aminotransferase (ALT), low density lipoprotein (LDL), serum creatinine (sCr), WBCs, monocytes, red blood cells (RBCs) and platelets (PLTs). However, the number of lymphocytes was significantly higher in the thrombolysis group compared to the patients treated with thrombectomy following thrombolysis (*p* = 0.03). Additionally, dyslipidaemia was most frequently observed in the thrombolysis group. Stroke severity at admission before treatment was higher in thrombolysis and thrombectomy patients versus two others groups (Table 1). Statistically significant differences were found between thrombolysis and thrombectomy group and thrombolysis group in NIHSS on the first day after treatment and 90-day mRS (Table 1).

### 3.2. Neutrophil-to-Lymphocyte Ratio (NLR) and Lymphocyte-to-Monocyte Ratio (LMR) in Treatment Subgroups

**Figure 1:**
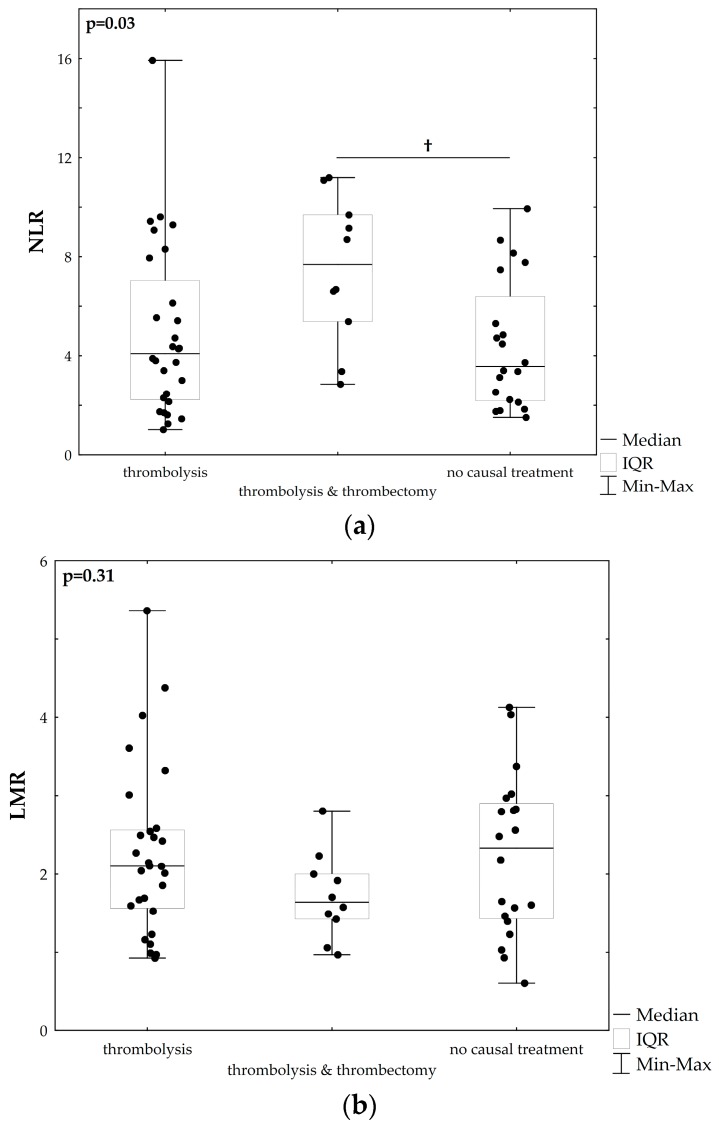
Comparison of neutrophil-to-lymphocyte ratio (NLR) (**a**) and lymphocyte-to-monocyte ratio (LMR) (**b**) in acute ischaemic stroke patients, stratified according to the type of treatment.

First, we compared NLR and LMR between the three patient subgroups. As shown in Figure 1a, NLR was significantly higher in patients treated with thrombolysis and thrombectomy compared to the patients who received thrombolysis and those without causal treatment (*p* = 0.03). In contrast, no significant differences in LMR were observed between the subgroups (*p* = 0.31, Figure 1b).

### 3.3. NLR, LMR and High-Sensitivity C-Reactive Protein (hs-CRP)

**Figure 2:**
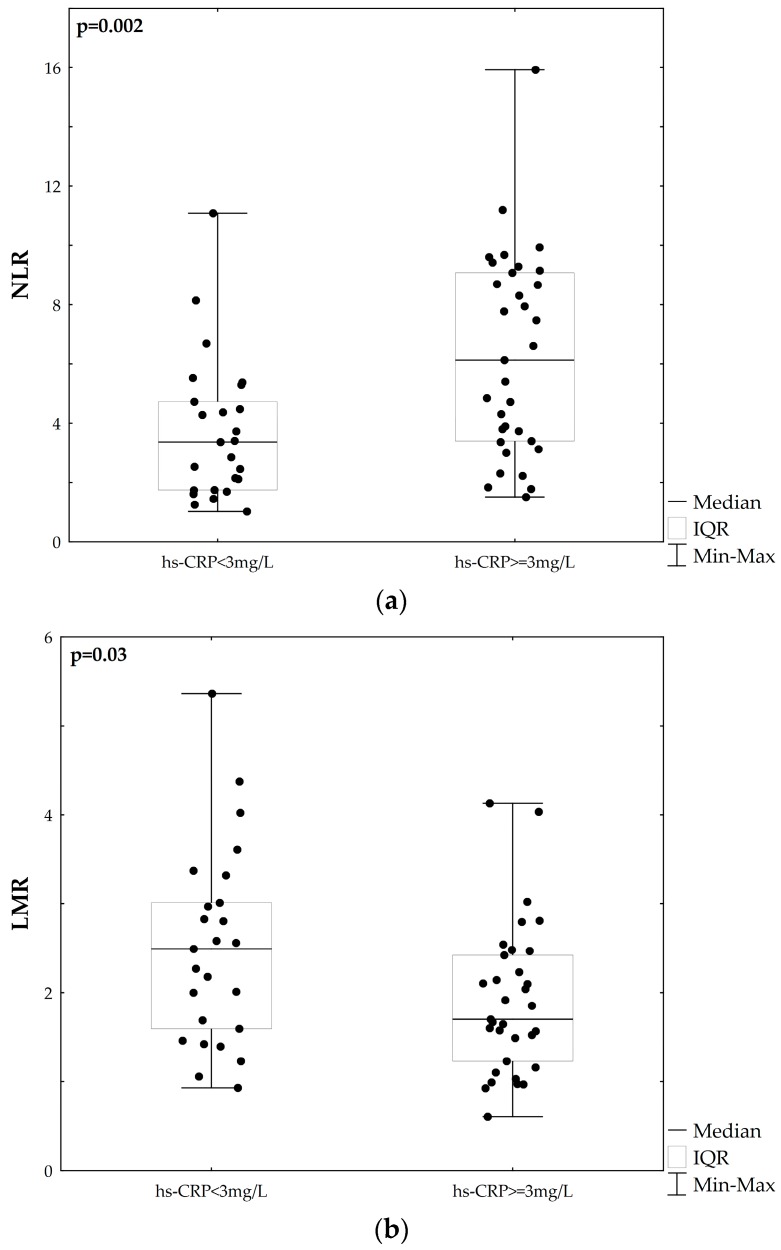
Comparison of NLR (**a**) and LMR (**b**) in acute ischaemic stroke patients, stratified according to the concentration of high-sensitivity C-reactive protein (hs-CRP).

To evaluate the relationship between NLR, LMR and hs-CRP, the patients were divided into two subsets: low hs-CRP subgroup, which represented patients with hs-CRP < 3 mg/L, and high hs-CRP subgroup, which represented patients with hs-CRP ≥ 3mg/L [16]. The concentrations of hs-CRP were measured at the same time as NLR and LMR. We found that NLR was higher, while LMR was lower in the patients from the high hs-CRP subgroup compared to those from the low hs-CRP subgroup (Figure 2).

### 3.4. NLR, LMR and the Severity of Stroke on the First Day after Treatment

Subsequently, our study group (n = 58) was divided based on the NIHSS score on the first day after treatment into two subgroups to compare those with a value ≤ 7 (n = 39) to those with a value >7 (n = 19) [17]. The obtained results showed a median value of NLR higher, in a statistically significant manner, in patients with an NIHSS score > 7 with respect to those with an NIHSS ≤ 7 (*p* = 0.0002). However, LMR was similar in both patient groups (*p* = 0.18). These results are summarised in Table 2.

### 3.5. NLR, LMR and Classifications of Stroke

The study group was divided according to the TOAST classification (Table 3), and no statistically significant differences between the five groups with respect to both NLR and LMR were observed. Subsequently, the study group was divided according to the OSCP classification, and the patients with TACI displayed a significantly higher NLR compared to those with LACI (*p* = 0.025, Table 4).

### 3.6. NLR, LMR and Ischaemic Stroke Comorbidities

Subsequently, NLR and LMR were quantified in patients divided according to the presence or absence of selected comorbidities, including smoking, CAD, diabetes mellitus, dyslipidaemia and AF. No significant differences in NLR and LMR were found in the comparison of patients who smoked and those having diabetes mellitus, dyslipidaemia and AF, with those without these comorbidities. Notwithstanding, LMR was significantly lower in stroke patients diagnosed with CAD (n = 10) compared to 48 patients without CAD (median of 1.31 versus 2.12, respectively, *p* = 0.028). This observation was not confirmed for NLR.

### 3.7. Correlation Analysis

The last step of our data analysis was the assessment of correlations between NLR, LMR, NIHSS on the first day after treatment and hs-CRP. NLR correlated positively with NIHSS (R = 0.5, *p* = 0.00001) and hs-CRP (R = 0.5, *p* = 0.0001). LMR correlated negatively with NIHSS (R = −0.3, *p* = 0.04) and hs-CRP (R = −0.3, *p* = 0.01).

## 4. Discussion

Our study showed that NLR was significantly higher in ischaemic stroke patients undergoing thrombolysis and thrombectomy compared to patients who were not treated causally. Intravenous thrombolysis, performed within ≤4.5 h from the onset of symptoms, or thrombectomy, which is mechanical clot removal that restores perfusion within ≤6 h from the onset of symptoms, are the standard of care in the management of ischaemic stroke. Our analysis indicates that high values of NIHSS in patients qualified for thrombectomy following thrombolysis may additionally suggest severe disability in these stroke victims. Acute brain ischaemia induces development of several molecular and cellular pro-inflammatory mechanisms involving nervous tissues as well as the vascular system. Many studies have documented infiltration of the ischaemic area by leukocytes and the relationship between the severity of inflammation, the size of cerebral infarction and the extent of neurological deficit [18,19]. Importantly, inflammation after stroke involves activation of microglia and astrocytes in the ischaemic area and migration of leukocytes from the blood to the tissues. Neutrophils are among the first cells that enter the ischaemic tissue already in the first few hours of cerebral infarction and contribute to the enlargement of the infarct area [20]. Further, monocytes accumulate in the brain tissue during the first 24 h of stroke, while lymphocytes appear as the last-within 24–48 h after the stroke [21,22]. Despite the progress in the diagnosis and treatment of stroke and the knowledge of pathophysiological processes during this disease, the relationship between the severity of inflammation, evidenced by increased NLR and decreased LMR, and the outcome of stroke treatment remains unexplained. Our results confirm that patients classified as eligible for thrombectomy following thrombolysis are those with severe clinical stroke and may be associated with an enhanced inflammatory reaction as described by high NLR. In contrast, we were not able to show any significant difference in LMR with respect to type of stroke therapy.

Patients who received two types of causal treatment presented with more severe strokes compared to the other two groups, as confirmed by significantly high NIHSS scores at admission and higher NIHSS scores 24 h after stroke diagnosis. The former group underwent thrombolytic treatment, with an additional thrombectomy considered necessary. Currently, the basic tool for thrombectomy is a stent retriever [23]. Its construction results from the need to obtain a device characterised by the best adhesion and penetrating the thromboembolic material inside the artery. Despite these facts, thrombectomy, as a mechanical intervention, is associated with cell damage. Perhaps, this might have been an additional factor that caused an increase in the inflammatory response in the form of a higher number of neutrophils and, consequently, a higher NLR in this group of patients. 

In our study, a significantly higher NLR was found in stroke patients who had higher hs-CRP (≥3 mg/L). Hs-CRP is an acute-phase protein that increases significantly in the case of tissue damage, infections and other inflammatory conditions [24]. Many studies have shown that hs-CRP is significantly higher in patients with ischaemic stroke and positively correlates with the severity of stroke [25,26,27]. Therefore, it is likely that NLR may become a substitute for hs-CRP in assessing the severity of inflammatory state.

In the presented study, median NLR was significantly higher in subjects who obtained more than 7 points according to the NIHSS on the first day after treatment. These analyses are similar to other studies performed in stroke patients [9,28,29]. Fang et al. presented a positive correlation between NLR and the severity of ischaemic stroke assessed using the NIHSS [25]. Our results were also comparable to those from a study by Xue et al. who reported that NLR was significantly related to the severity of stroke at admission [8]. Moreover, it has been reported that patients with NLR ≥ 3.3 had over 2 times higher risk of hospital mortality compared to those with NLR < 3.2. Several clinical papers have also proposed that NLR is useful to predict short- and long-term mortality in patients after ischaemic stroke [30,31,32]. Our pilot study confirms previous observations on the utility of NLR in the diagnosis of stroke severity. Future research should therefore be directed towards to the possible of clinical application of NLR.

The aetiology of ischaemic stroke can be varied, which is reflected in the pathophysiological classification of TOAST. However, no differences in the NLR values in groups of patients with ischaemic stroke of different aetiologies were found in our study. This is in line with the literature data, in which it has been noticed that similar pathophysiological changes occur in the ischaemic cerebral tissue in different types of stroke [33]. Additionally, in the present study, we analysed the value of NLR in individual anatomic stroke groups and found that NLR was higher in a statistically significantly manner in the group of patients with TACI stroke in comparison to LACI. It is consistent with observations by other researchers that early neutrophilia is associated with larger infarct volumes [34].

It is also interesting to note that previous clinical studies have demonstrated that NLR may be useful for predicting clinical outcome in patients with haemorrhagic stroke [35]. The studies performed by one Italian group have clearly shown that patients with poor outcomes had higher NLR [36,37,38]. In light of these results, consideration should be given to the introduction of this indicator in routine neurological diagnostics. Moreover, these observations are important from a pathophysiological perspective, linking inflammation with the etiopathogenesis of both stroke types.

This study has two main limitations; firstly, due to the single-centre nature of the present study and the use of restrictive inclusion and exclusion criteria, the sample size was relatively small. Nevertheless, we were able to show a relationship between NLR, stroke severity, and treatment modalities of ischemic stroke patients. Secondly, control individuals without ischemic stroke were not enrolled; this was because the main aim of this study was the evaluation of the link between treatment strategies, NLR, and LMR. Moreover, repeated measurements of NLR and LMR at different time points could improve the reliability of our results. Without a doubt, further studies are warranted to clarify these observations.

## 5. Conclusions

In conclusion, the results of this study show a significant association between higher NLR, type of stroke treatment and stroke severity. Thus, NLR introductions into everyday practice can facilitate risk assessment and improve the prognosis of patients in the acute phase of ischaemic stroke. These preliminary results could be also used for future research.

## Figures and Tables

**Table 1:** Demographic and clinical data of the study group stratified according to type of treatment.

Parameter (unit)		Whole Cohort (n = 58)	Type of Treatment			
			Thrombolysis (n = 28)	Thrombolysis and Thrombectomy (n = 10)	No Causal Treatment (n = 20)	*p*
Age (years)		67 (60–77)	62 (45–75)	68 (62–77)	71 (64–79)	0.10
SEX—male (%)		29 (50)	12 (43)	5 (50)	12 (60)	0.50
BMI (kg/m^2^)		28 (24–31)	27 (23–31)	27 (25–33)	29 (26–30)	0.85
SBP (mmHg)		130 (120–140)	130 (130–135)	130 (120–160)	130 (125–140)	0.99
DBP (mmHg)		80 (80–80)	80 (80–80)	80 (80–90)	80 (73–80)	0.03
LVEF (%)		63 (55–67)	65 (55–67)	53 (45–60)	65 (53–69)	0.09
TOAST n (%)	LAA	13 (22)	2 (7)	5 (50)	6 (30)	0.14
	SVO	5 (9)	4 (14)	1 (10)	0	
	CE	12 (21)	6 (21)	2 (20)	4 (20)	
	SOE	4 (7)	3 (11)	0	1 (5)	
	SUE	24 (41)	13 (46)	2 (20)	9 (45)	
OSCP	LACI	18 (31)	12 (43)	1 (10)	5 (25)	0.08
	PACI	24 (41)	12 (43)	4 (40)	8 (40)	
	TACI	5 (9)	0	3 (30)	2 (10)	
	POCI	11 (19)	4 (14)	2 (20)	5 (25)	
TICI	TICI 0			1 (10)		-
	TICI 2b			6 (60)		
	TICI 3			3 (30)		
Stroke scales	NIHSS at admission before treatment	7 (4–13)	6 (4–9) ^a^	14 (11–15) ^a,b^	5 (4–11) ^b^	0.006
	NIHSS on the first day after treatment	4 (3–11)	3 (2–6) ^a^	15 (9–18) ^a^	5 (3–9)	0.002
	mRS at admission before treatment	0 (0–0)	0 (0–0)	0 (0–0)	0 (0–1)	0.45
	90–day mRS	1 (1–3)	1 (1–2) ^a^	4 (1–5) ^a^	1 (1–3)	0.02
	ASPECTS at admission	10 (9–10)	10 (9–10)	6 (5–9)	10 (10–10)	0.03
AST (U/L)		18 (16–24)	18 (16–24)	22 (18–26)	18 (14–23)	0.33
ALT (U/L)		17 (13–22)	16 (13–23)	17 (17–23)	17 (11–22)	0.48
sCr (mg/dL)		0.85 (0.71–1.08)	0.86 (0.72–1.01)	0.79 (0.66–1.11)	0.85 (0.70–1.11)	0.95
LDL (mg/dL)		102 (74–138)	104 (74–140)	89 (77–119)	102 (74–132)	0.89
CBC	WBCs [10^3^/μL]	9.88 (8.31–11.32)	10.43 (8.45–12.83)	10.36 (8.64–13.46)	8.97 (7.66–10.39)	0.22
	NEUTs [10^3^/μL]	7.08 (5.02–9.04)	7.60 (4.68–9.13)	8.85 (6.51–12.49)	6.52 (5.34–6.94)	0.08
	LYMPHs [10^3^/μL]	1.69 (1.20–2.22)	1.91 (1.36–2.43) ^a^	1.15 (0.87–1.45) ^a^	1.62 (1.21–2.19)	0.03
	MONOs [10^3^/μL]	0.85 (0.64–1.02)	0.93 (0.73–1.13)	0.85 (0.51–1.03)	0.79 (0.62–0.92)	0.36
	RBCs [10^6^/μL]	4.41 (4.10–4.87)	4.37 (4.06–4.75)	4.35 (3.99–4.52)	4.66 (4.18–4.90)	0.58
	PLTs [10^3^/μL]	232 (189-261)	240 (208-262)	221 (164-333)	215 (184-253)	0.66
Current smokers, n (%)		18 (31)	8 (29)	2 (20)	8 (40)	0.50
Medical history, n (%)	CAD	10 (17)	3 (11)	4 (40)	3 (15)	0.10
	Previous AIS	12 (21)	3 (11)	2 (20)	7 (35)	0.12
	Hypertension	45 (78)	21 (75)	7 (70)	17 (85)	0.58
	Diabetes mellitus	16 (28)	8 (29)	3 (30)	5 (25)	0.95
	Dyslipidemia	32 (55)	20 (71)	3 (30)	9 (45)	0.04
	AF	11 (19)	5 (18)	4 (40)	2 (10)	0.14

Data are expressed as median (interquartile range, IQR) or number (%) of patients. *p* values were determined using the Pearson’s chi-square tests (categorical variables) or the Kruskal Wallis tests (continuous variables). ^a,b^ *p* value obtained by pairwise comparison < 0.05; Abbreviations are n: Number, BMI: Body mass index, SBP: Systolic blood pressure, DBP: Diastolic blood pressure, LVEF: Left ventricular ejection fraction, TOAST: Trial of ORG 10172 in Acute Stroke Treatment, LAA: Large-artery atherosclerosis, SVO: Small vessel occlusion, CE: Cardioembolism, SOE: Stroke of other determined etiology, SUE: Stroke of undetermined etiology, OSCP: Oxfordshire Community Stroke Project, LACI: Lacunar circulation infarcts, PACI: Partial anterior circulation infarcts, TACI: Total anterior circulation infarcts, POCI: Posterior circulation infarcts, TICI: Thrombolysis in cerebral infarction, NIHSS: National Institutes of Health Stroke Scale, mRS: Modified Rankin Scale, ASPECTS: Alberta stroke programme early CT score, AST: Aspartate aminotransferase, ALT: Alanine aminotransferase, sCr: Serum creatinine, LDL: Low-density lipoprotein, CBC: Complete blood count, WBCs: White blood cells, NEUTs: Neutrophils, LYMPHs: Lymphocytes, MONOs: Monocytes, RBCs: Red blood cells, PLTs: Platelets, CAD: Coronary artery disease, AIS: Acute ischemic stroke, and AF: Atrial fibrillation.

**Table 2:** Comparison of neutrophil-to-lymphocyte ratio and lymphocyte-to-monocyte ratio between subgroups based on NIHSS on the first day after treatment.

Parameter	NIHSS ≤ 7 (n = 39)	NIHSS > 7 (n = 19)	*p*
NLR	3.74 (2.12–5.30)	8.68 (3.73–9.61)	**0.0002**
LMR	2.18 (1.53–2.83)	1.89 (1.23–2.23)	0.18

Data are expressed as median (interquartile range, IQR); *p* values were determined using Mann–Whitney *U* tests. Abbreviations are NIHSS: National Institutes of Health Stroke Scale, NLR: Neutrophil-to-lymphocyte ratio, and LMR: Lymphocyte-to-monocyte ratio.

**Table 3:** Comparison of neutrophil-to-lymphocyte ratio and lymphocyte-to-monocyte ratio between subgroups based on Trial of Org 10172 in Acute Stroke Treatment (TOAST) classification.

Parameter	SUE (n = 24)	LAA (n = 13)	CE (n = 12)	SOE (n = 4)	SVO (n = 5)	*p*
NLR	3.85 (2.34–6.65)	4.85 (2.31–9.15)	6.37 (4.22–9.36)	3.66 (2.58–5.89)	3.41 (1.70–4.72)	0.23
LMR	2.19 (1.43–2.81)	2.00 (1.58–2.56)	1.63 (1.33–2.14)	2.02 (1.27–3.04)	2.49 (1.59–2.58)	0.77

Data are expressed as median (interquartile range, IQR); *p* values were determined using Kruskal–Wallis tests. Abbreviations are NLR: Neutrophil-to-lymphocyte ratio, LMR: Lymphocyte-to-monocyte ratio, LAA: Large-artery atherosclerosis, SVO: Small vessel occlusion, CE: Cardioembolism, SOE: Stroke of other determined etiology, and SUE: Stroke of undetermined etiology.

**Table 4:** Comparison of neutrophil-to-lymphocyte ratio and lymphocyte-to-monocyte ratio between subgroups based on Oxfordshire Community Stroke Project (OSCP) classification.

Parameter	LACI (n = 18)	PACI (n = 24)	POCI (n = 11)	TACI (n = 5)	*p*
NLR	4.34 ^a^ (1.70–5.53)	4.31 (2.66–8.87)	3.40 (2.53–6.61)	9.93 ^a^ (9.15–11.09)	0.025
LMR	2.38 (1.57–2.97)	2.10 (1.33–2.55)	1.69 (1.46–2.48)	1.70 (1.58–2.00)	0.81

Data are expressed as median (interquartile range, IQR); *p* values were determined using Kruskal–Wallis tests; ^a^ *p* value obtained by pairwise comparison < 0.05. Abbreviations are NLR: Neutrophil-to-lymphocyte ratio, LMR: Lymphocyte-to-monocyte ratio, LACI: Lacunar circulation infarcts, PACI: Partial anterior circulation infarcts, TACI: Total anterior circulation infarcts, and POCI: Posterior circulation infarcts.
